# Supplementary material for: A cost-effective alkaline polysulfide-air redox flow battery enabled by a dual-membrane cell architecture
Source: Nat Commun. 2022 May 2;13:2388. doi: 10.1038/s41467-022-30044-w (PMC9061742; doi:10.1038/s41467-022-30044-w)
Supplement: Supplementary file 1 — Supplementary Information [file 41467_2022_30044_MOESM1_ESM.pdf]

Supplementary Information

A cost-effective alkaline polysulfide-air redox flow battery enabled by a dual-membrane cell architecture

Yuhua Xia [1,#], Mengzheng Ouyang [1,#,*], Vladimir Yufit [1,2], Rui Tan [3], Anna Regoutz [4], Anqi Wang [3], Wenjie Mao [3], Barun Chakrabarti [1,5], Ashkan Kavei [1,6], Qilei Song[3], Anthony R. Kucernak [6,7] and Nigel P. Brandon[1, 6]

[1] Department of Earth Science and Engineering, Imperial College London, London SW7 2AZ, United Kingdom
[2] Addionics Ltd., Imperial White City Incubator, 80 Wood Lane, London, W12 0BZ, United Kingdom
[3] Department of Chemical Engineering, Imperial College London, London SW7 2AZ, United Kingdom
[4] Department of Chemistry, University College London, 20 Gordon Street, London, WC1H 0AJ, United Kingdom
[5] WMG, Warwick Electrochemical Engineering Group, Energy Innovation Centre, University of Warwick, Coventry, CV4 7AL, United Kingdom
[6] RFC Power Ltd., Imperial White City Incubator, 80 Wood Lane, London, W12 0BZ, United Kingdom
[7] Department of Chemistry, Imperial College London, London SW7 2AZ, United Kingdom

[#] These authors contribute equally
*corresponding author, Email: m.ouyang15@imperial.ac.uk

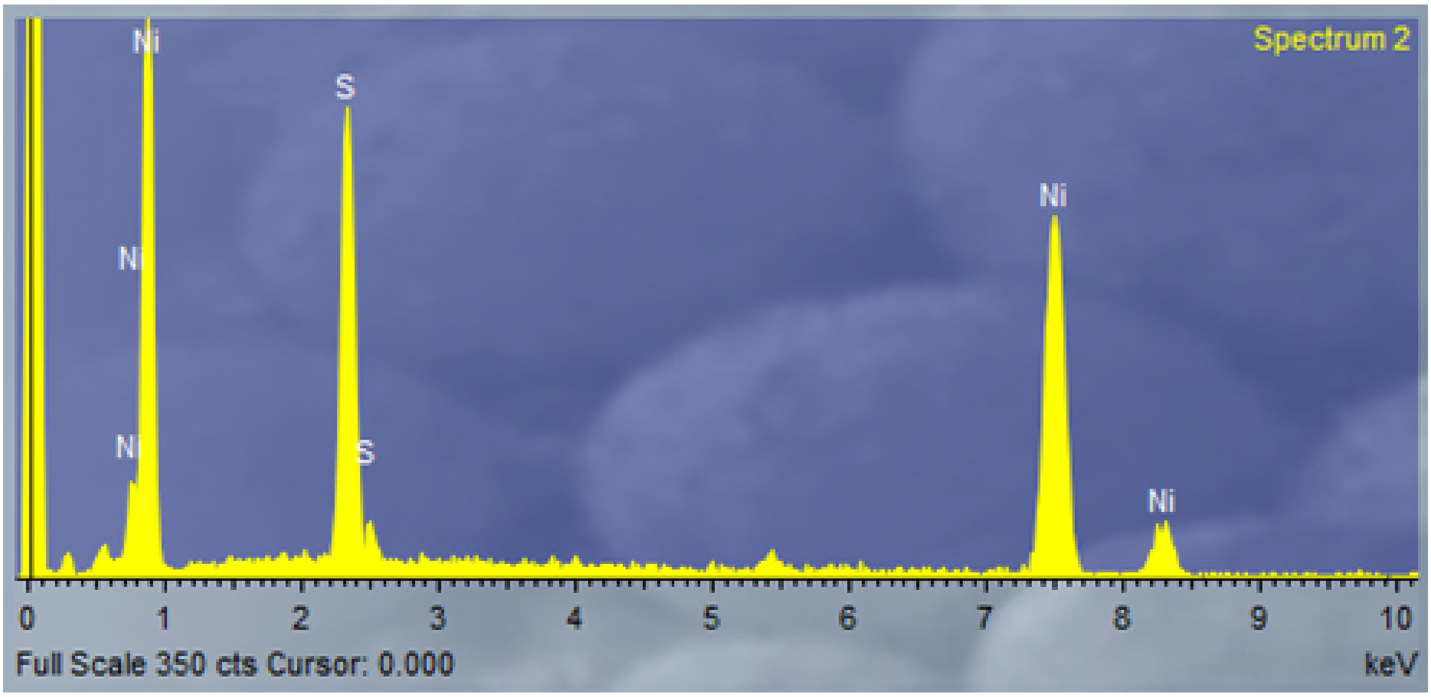

Supplementary Figure 1. EDX spectrum of the sulfidised Ni foam. There are two major elements existing on the surface of the sulfidised Ni foam, namely Ni and S. This indicates that the species formed on the sulfidised Ni foam surface was likely NiSx.

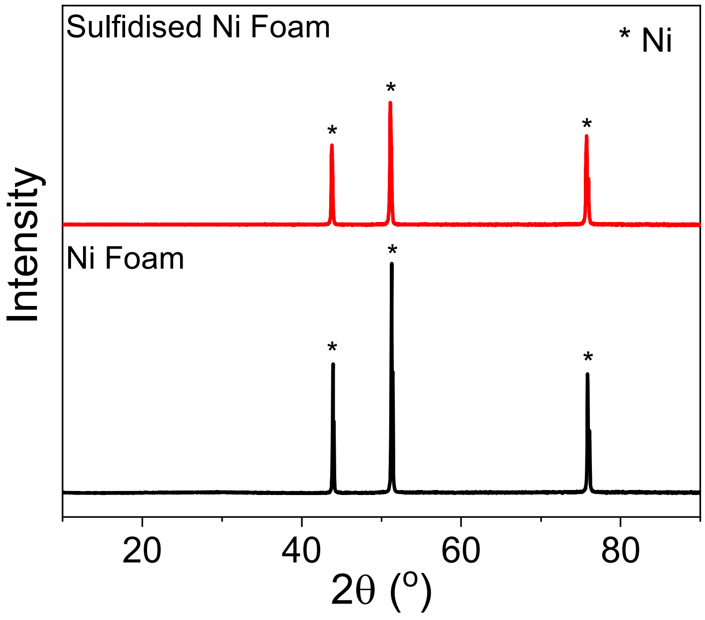

Supplementary Figure 2. XRD patterns of the as-received and sulfidised Ni foams

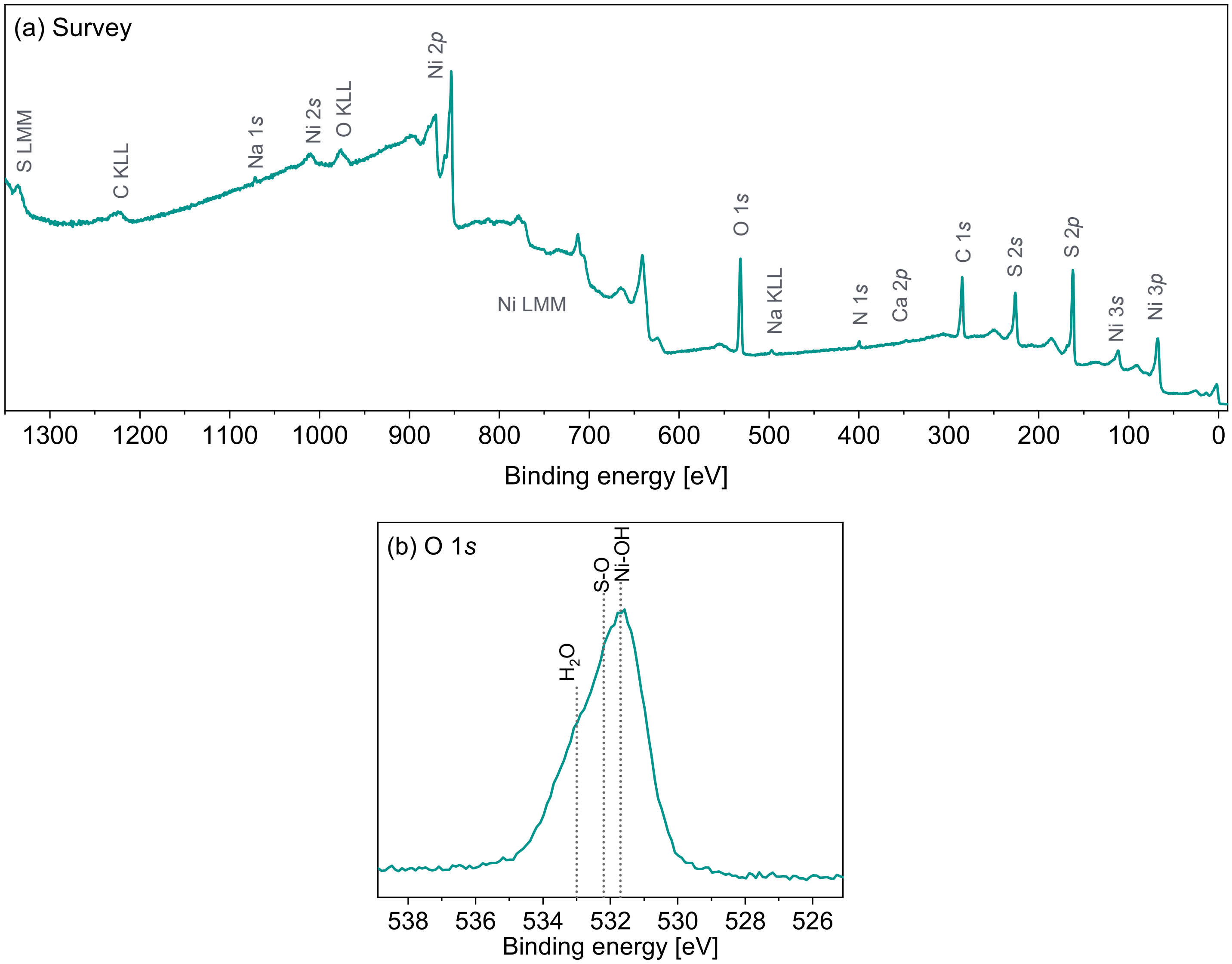

Supplementary Figure 3. XPS spectra of the sulfidised Ni foam, including (a) survey, (b) Ni 2*p*, (c) S 2*p*, and (d) O 1s spectra. All major lines including Auger lines are indicated in the survey scan.

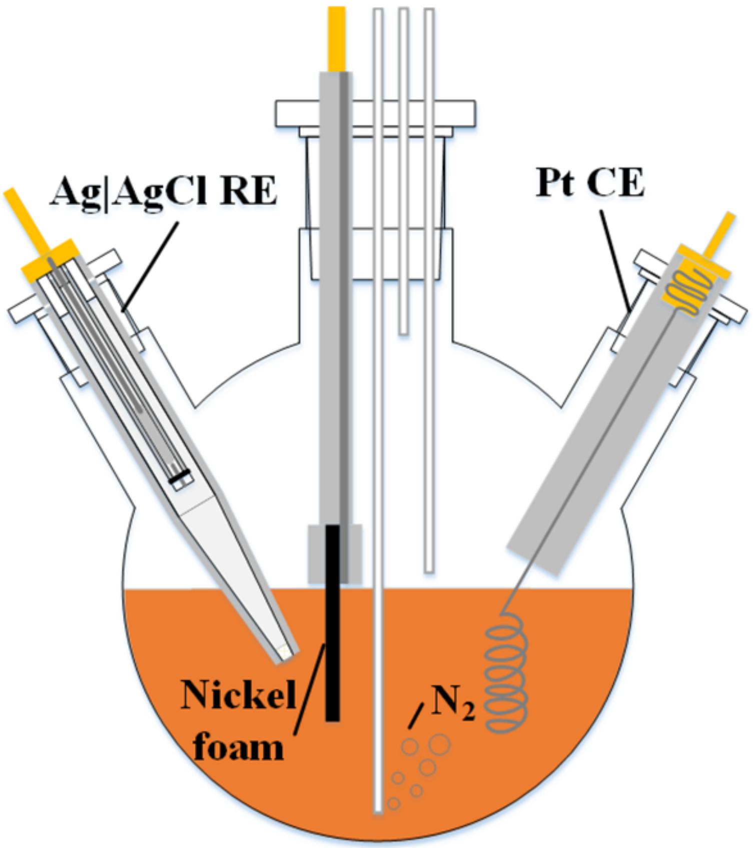

Supplementary Figure 4. A schematic view of the in-house constructed three-electrode cell setup, with a nickel foam sheet working electrode, a Pt counter electrode and an Ag|AgCl (3 M KCl electrolyte saturated with AgCl) reference electrode.

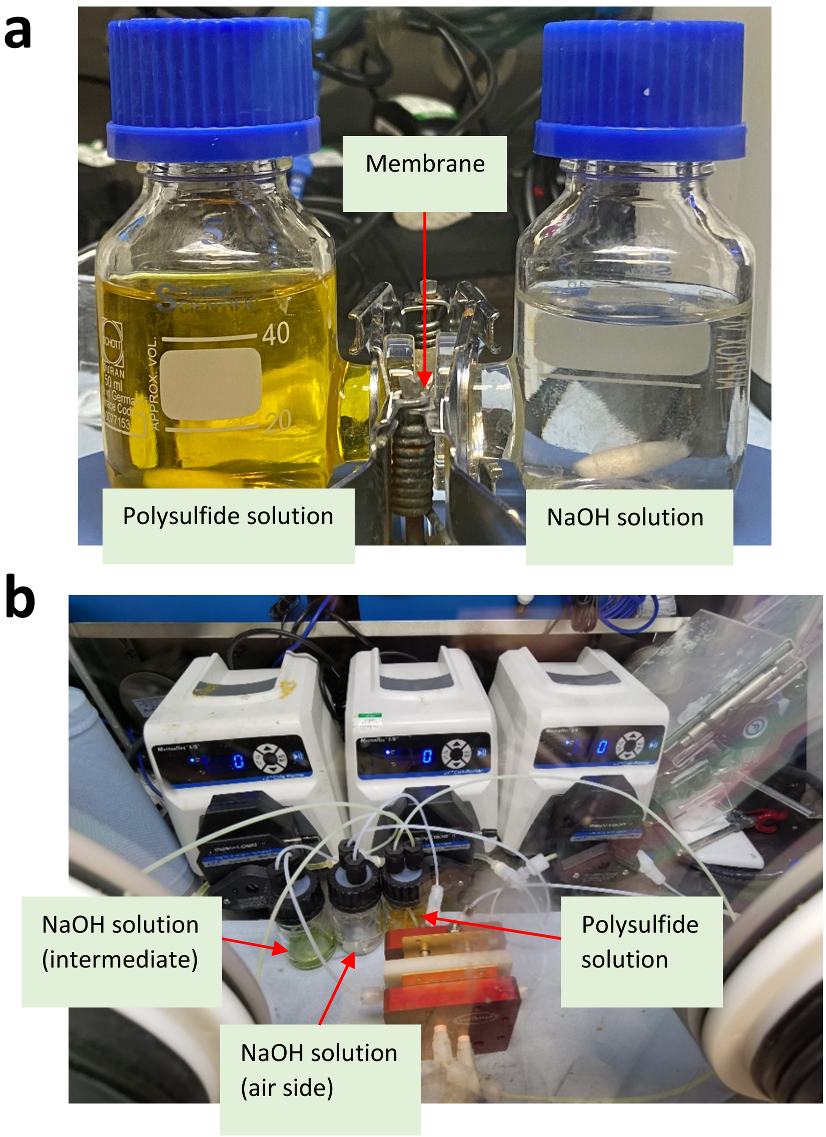

Supplementary Figure 5. Photographic pictures of polysulfide crossover determination setups: (a) H-cell setup for single-membrane structure and for (b) redox-flow battery setup for dual-membrane structure

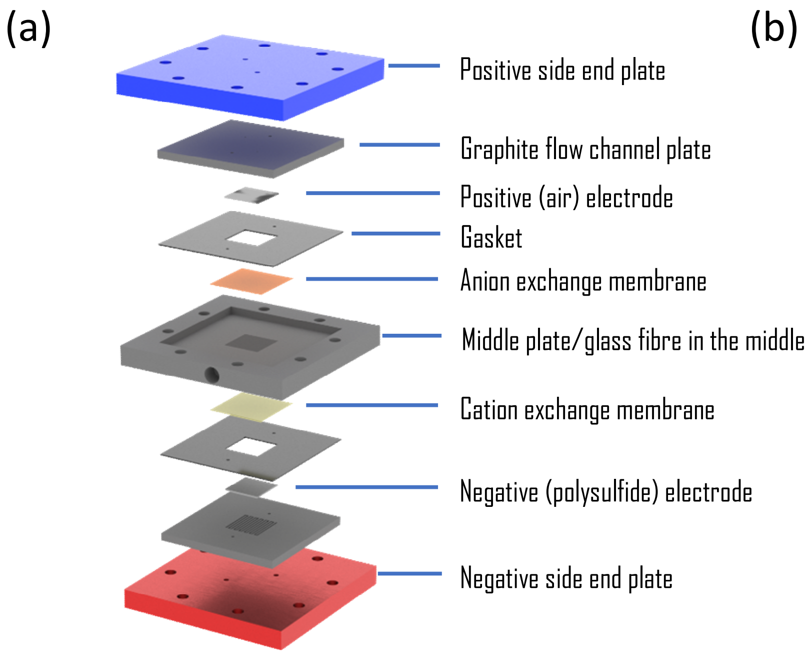

Supplementary Figure 6. The exploded views of the single-cell reactor for the alkaline PSA RFB with: (a) double-membrane cell design and (b) single-membrane cell design.

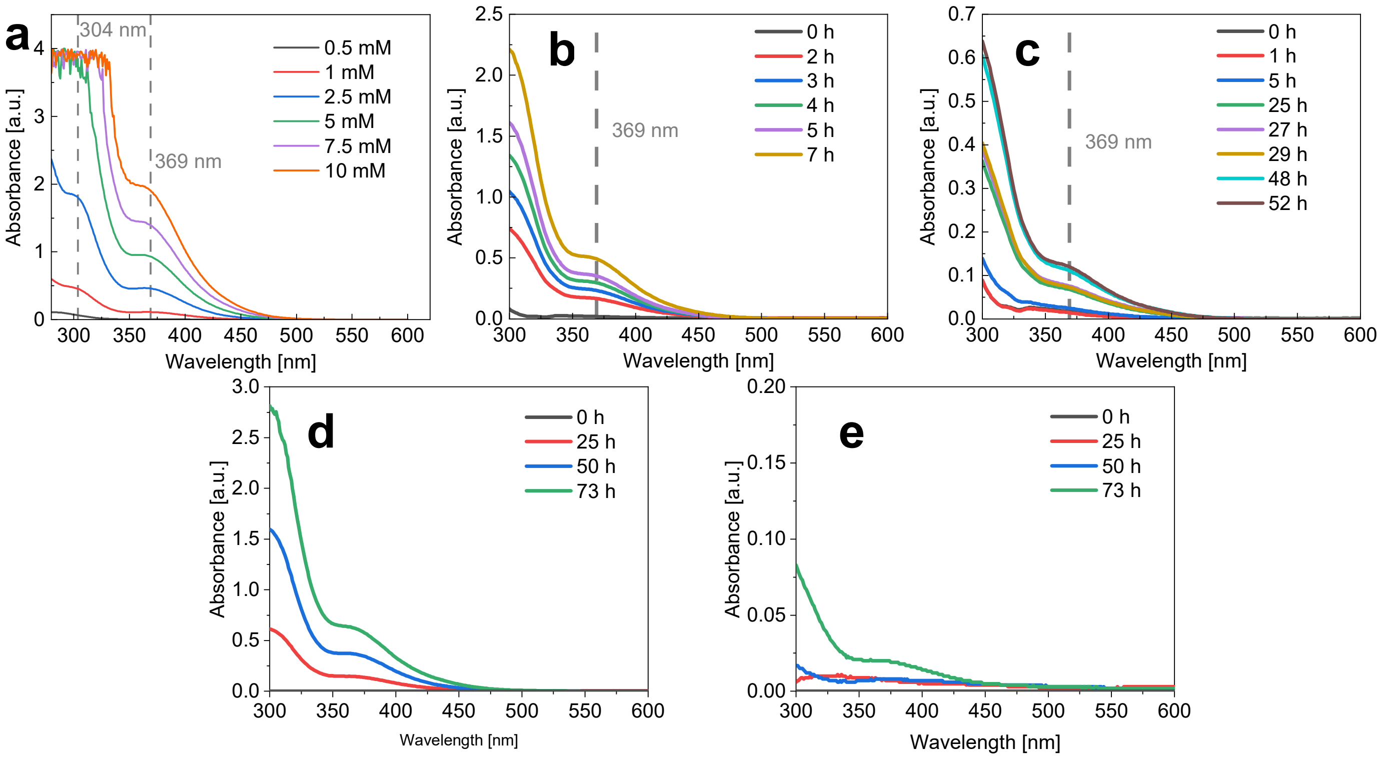

Supplementary Figure 7. UV-Vis spectrum of (a) standard Na_2S_2 solution of concentrations ranging from 0.5 mM to 10 mM, (b) the permeate solution in the H-Cell setup assembled with AEM after various permeation times, (c) the permeate solution in the H-Cell setup assembled with CEM after various times, (d) the intermediate solution in the dual membrane setup after various time, (e) the solution after flushing air side of the dual membrane setup after various times.

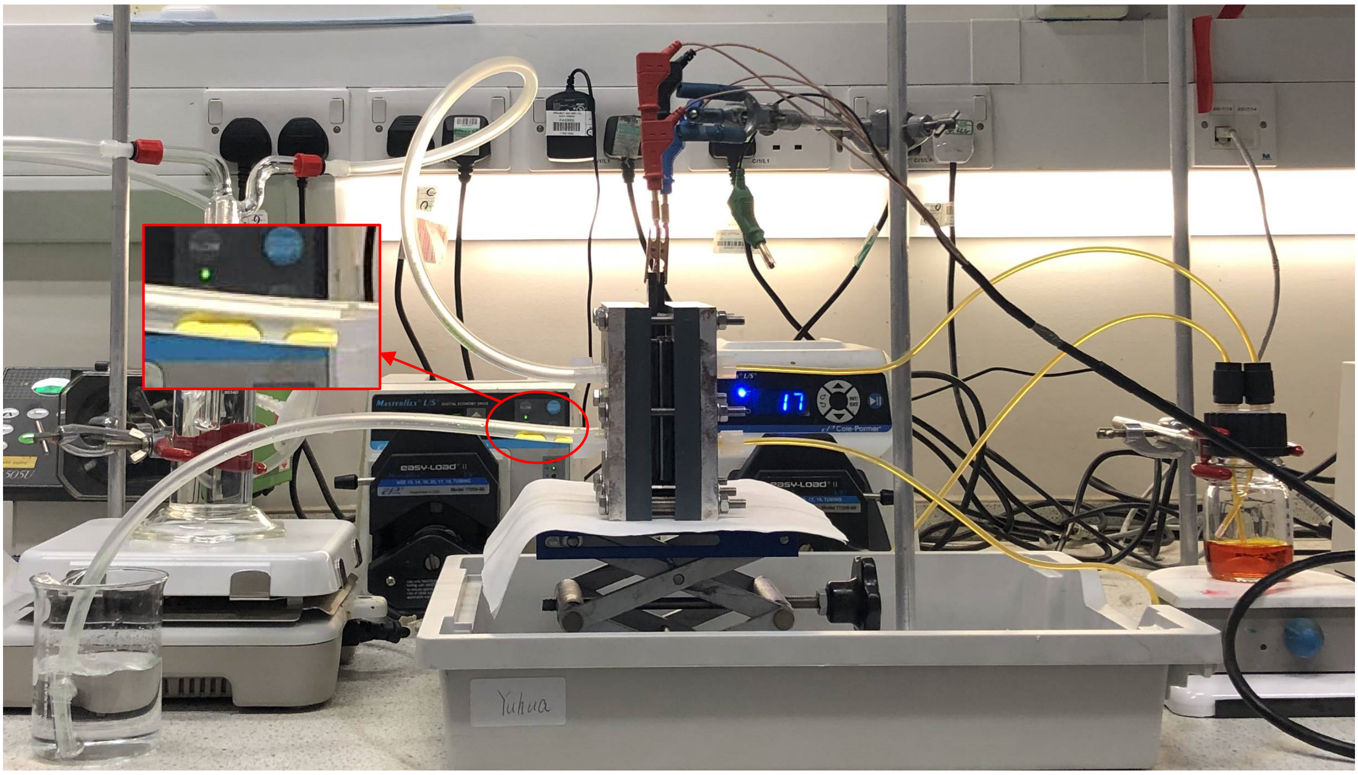

Supplementary Figure 8. A photo of the experimental setup of a 5 cm^2 single-AEM alkaline PSA RFB. The red circle indicates the location of the yellow solution present at the outlet of the air side half-cell, which clearly shows the existence of polysulfide species.

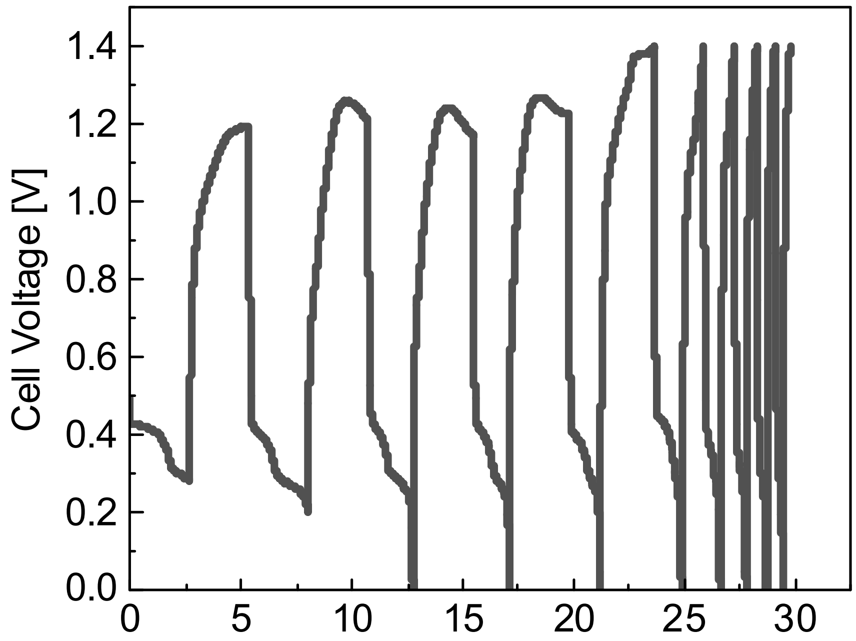

Supplementary Figure 9. Cell voltage of the single-AEM-structured alkaline PSA RFB over 10 cycles

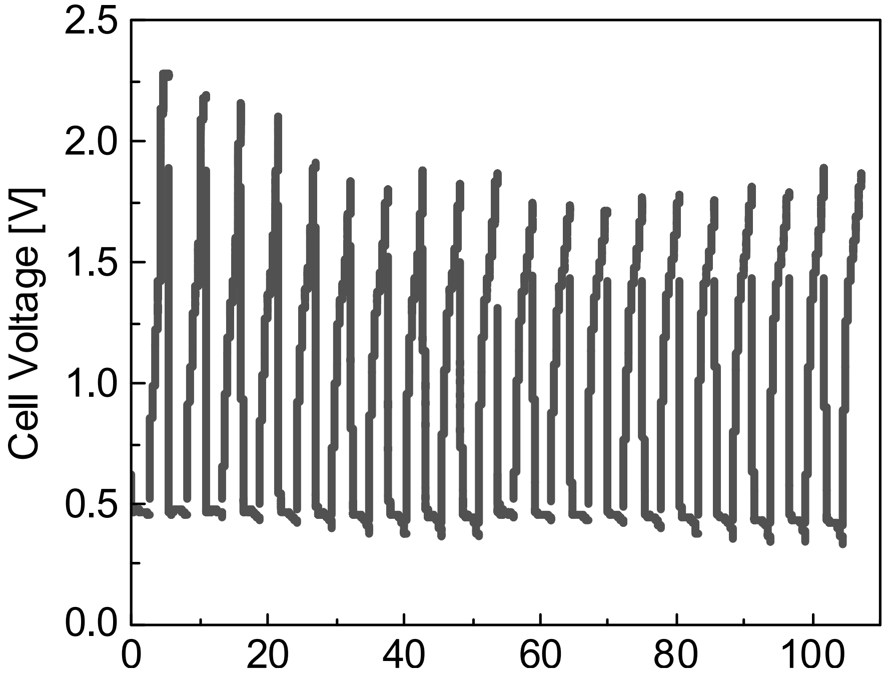

Supplementary Figure 10. Cell voltage of the single-CEM-structured alkaline PSA RFB over 20 cycles

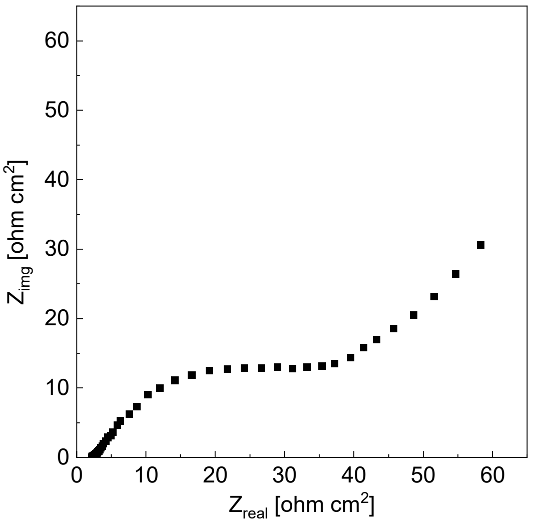

Supplementary Figure 11. Nyquist plot of the double-membrane PSA RFB. The EIS measurement was performed at OCV and 100 SOC, before the first cycle.

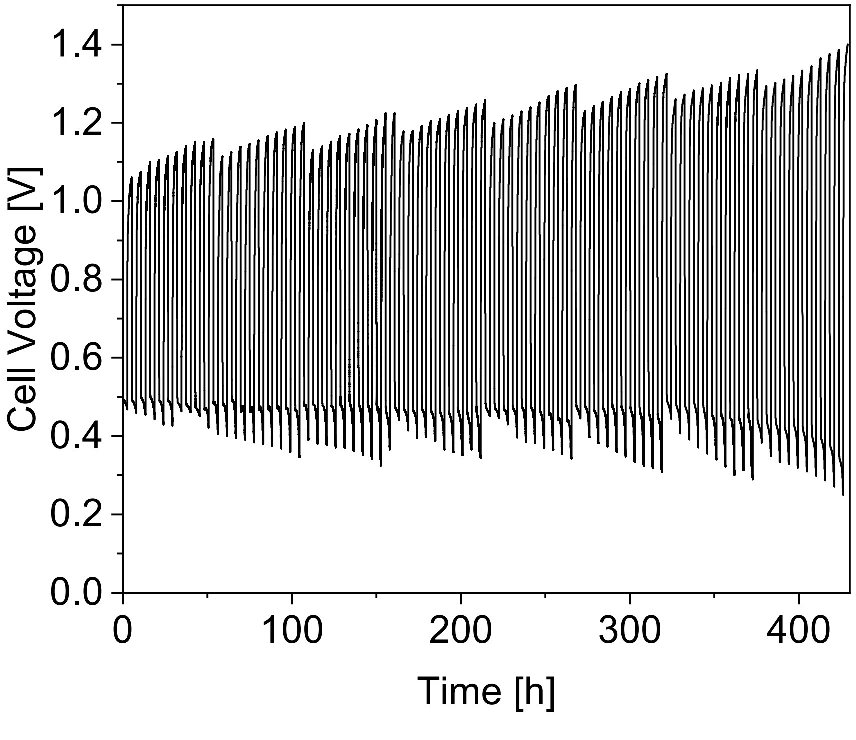

Supplementary Figure 12. Cell Voltage of the double-membrane-structured alkaline PSA RFB over 80 cycles

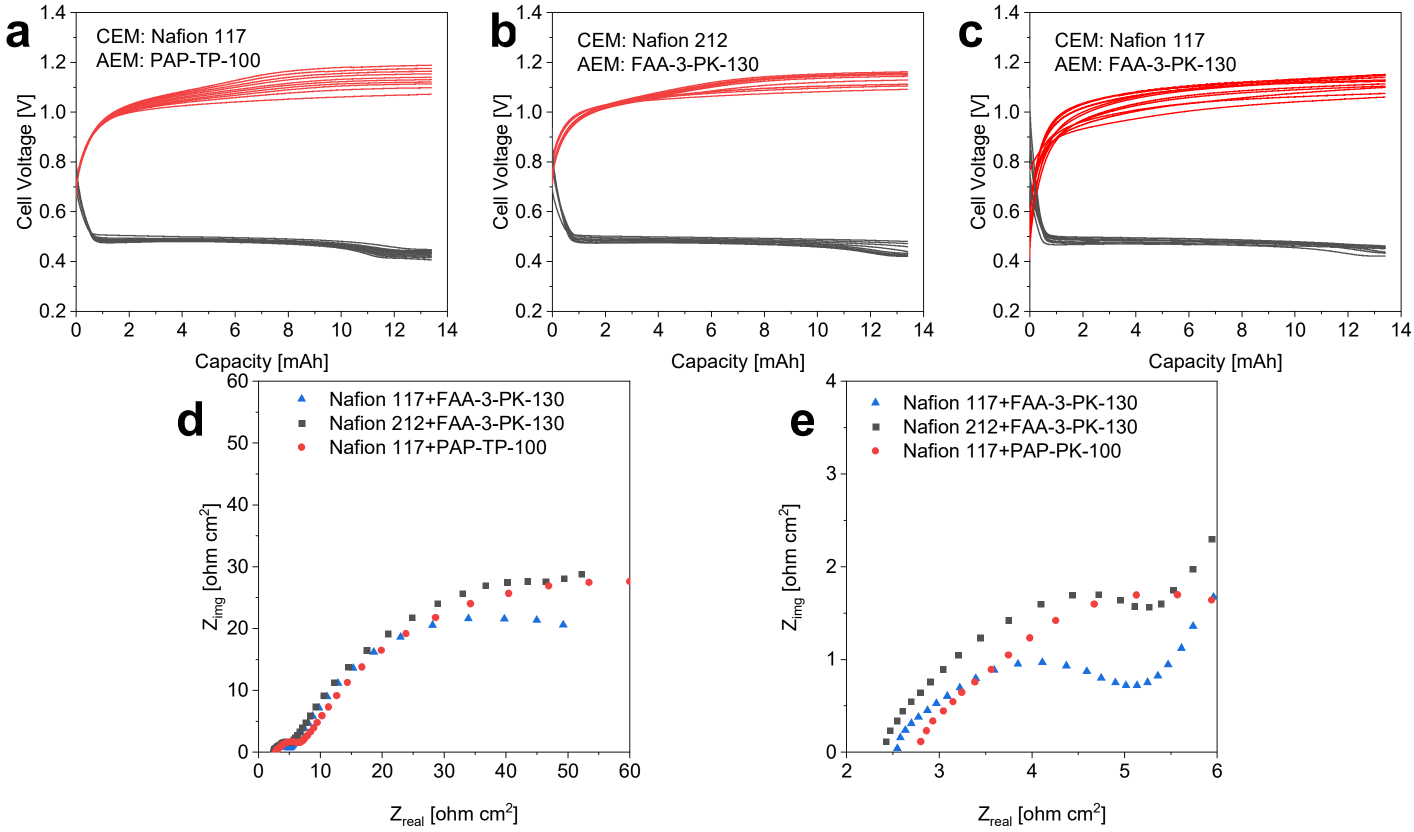

Supplementary Figure 13. EIS and 10 cycles of dual-membrane polysulfide-air redox flow batteries using different membrane combinations: First 10 cycles of PSA RFB using: a. Nafion 117 as CEM, PAP-TP-100 as AEM. b. Nafion 212 as CEM, FAA-3-PK-130 as AEM. c. Nafion 117 as CEM, FAA-3-PK-130 as AEM. d. EIS and e. magnified EIS measurements of PSA RFB with different membranes at OCV, 100 SOC before the first cycle.

Supplementary Table 1. Exchange current densities and Tafel slopes of nickel foams before and after pre-treatments in the 1 m Na_2S_2 / 1 m NaOH electrolyte

	Cathodic i_0 (mA cm^{-2})	Anodic i_0 (mA cm^{-2})	Average i_0 (mA cm^{-2})	Cathodic b (mV $decade^{-1}$)	Anodic b (mV $decade^{-1}$)
As-received Ni foam	0.66	3.75	2.21	252	196
Sulfidised Ni foam	8.20	6.09	7.15	260	253

Supplementary Table 2. Full-cell and half-cell potentials of the alkaline PSA RFB during the 1st and 30th cycle

	Full-cell		Polysulfide-side		Air-side	
	Discharging	Charging	Discharging	Charging	Discharging	Charging
Averaged potential at the 1st cycle (V)	0.487	0.992	-0.399	-0.582	0.088	0.410
Averaged potential difference between charge and discharge at the 1st cycle (V)	0.505		-0.183		0.322	
Averaged potential at the 30th cycle (V)	0.327	1.305	-0.381	-0.617	-0.054	0.688
Averaged potential difference between charge and discharge at the 30th cycle (V)	0.978		-0.236		0.634	
Averaged potential change over 30 cycles (V)	-0.16	0.313	0.018	-0.035	-0.142	0.278

- The average potential is calculated by averaging the potential recorded along the time axis of the charging or discharging process, respectively.

Supplementary Table 3. The power cost of the double-membrane-structured alkaline PSA RFB

**Stack component**	**Cost (US$/m²)**	**Cost (US$/kW)**	**Reference**
Ni foam	18.00	309.81	[1]
MnO_2	0.06	1.03	[1]
GDL	4.93	84.85	[2]
AEM	20.00	344.23	[2]
CEM	20.00	344.23	[2]
Bipolar plate	30.00	516.35	[2]
Frame, seals, and manifolds	2.00	34.42	[3]
Total		1635	

Supplementary Table 4. The power cost of the previously reported state-of-the-art PSA RFB using acidic catholyte

Stack component	Cost (US$/m^2)	Cost (US$/kW)	Reference
Pt	65.86	1291.39	[4]
GDL	4.93	96.67	[5]
IrO_2	225.02	4412.13	[4]
Titanium plate	22.55	442.16	[3]
Stainless steel	5.90	115.69	[3]
SSE separator	100.00	1960.78	[3]
Frame, seals, and manifolds	2.00	39.22	[3]
Total		8358	

Supplementary Table 5. The chemical cost of the double-membrane-structured alkaline PSA RFB, with 5 M total dissolved S content

	Chemical	Bulk price (US$/kg)	Concentration (M)	Cost breakdown (US$/kWh)
$C_{anolyte}$	Na_2S	0.26	2.5	2.23
	S	0.05	2.5	0.11
$C_{catholyte}$	NaOH	0.2	1	0.21
C_{energy}	-	-	-	2.54

Supplementary Table 6. The chemical cost of the previously reported state-of-the-art PSA RFB using acidic catholyte and Li^+ as the working ion, with 5 M total dissolved S content

	Chemical	Price (US$/kg)	Concentration (M)	Cost breakdown (US$/kWh)
$C_{anolyte}$	Li_2SO_4	1.00	2.6	4.51
	H_2SO_4	0.10	0.1	0.02
$C_{catholyte}$	Li_2S	1	1.25	2.81
	S	0.05	3.75	0.42
	LiOH	1.77	3	2.01
C_{energy}	-	-		9.77

Supplementary Table 7. Comparison of key performance indicators in this work with PSA systems in the literature

Anolyte	Anode	Catholyte	Cathode	Membrane	Active Area (cm^2)	OCV (V)	Peak power density (mW/cm^2)	Cycling temperature (°C)	Cycling current density (mA/cm^2)	Round-trip energy efficiency (%)	Energy cost (C_{energy}, US$/kWh)	Power cost (C_{power}, US$/kW)	Refernce
0.1-1 M Na_2S_2 + 1 M NaOH	Sulfidised Ni foam	1 M NaOH	MnO_2/C@gold-coated Ni mesh (Gaskatel GmbH)	Nafion 117, FAA-3-PK-130	5 cm^2	~0.81	5.81 (55 °C)	25	1-5	40 (80 cycles at 1 mA cm^{-2})	2.54	1635	This work
1 M Li_2S_4 + 1 M LiOH	Sulfidised Ni mesh	0.5 M Li_2SO_4 + 0.5 M H_2SO_4	Pt/C@GDL, IrO_2@platinised Ti screen	LISICON (50-150 µm)	-	~1.35	5.1 (55 °C, 50 µm LiSICON)	25	0.325-2	43 (40 cycles at 0.325 mA cm^{-2})	9.77	8358	[3]
1 M Na_2S_2 + 0.1 M NaOH	CuS/GDL (1.75 mg cm^{-2})	0.1 M H_3PO_4 + 1 M NaH_2PO_4	Pt/C@GDL (0.85 mg cm^{-2}), IrO_2@Ti mesh	NASICON	-	-	-	-	0.5	-	-	-	[6]

Supplementary Note 1 XPS spectroscopy on sulfidised nickel foam

Supplementary Figure 4 presents the XPS survey spectrum and high-resolution spectra of the Ni 2*p*, S 2*p* and O 1*s* core levels of the sulfidised Ni foam. From the survey spectrum, the core level and Auger peaks from Ni and S as well as a range of surface contamination peaks were observed, as shown in Supplementary Figure 4 (a). Considerable amounts of both C and O are found as well as smaller levels of Na, Ca and N are found in the survey spectrum, which are not surprising given the preparation and handling of the samples in liquid environments and air. In order to investigate the chemical state of the sample further, high resolution spectra of the Ni 2*p*, S 2*p* and O 1*s* core levels were also collected, as shown in Supplementary Figure 4 (b-d).

Supplementary Figure 4 (b) shows the Ni 2*p* core level including the Ni $2p_{3/2}$ and Ni $2p_{1/2}$ regions, which exhibit a typical spin orbit splitting (SOS) of 17.4 eV. The strong contribution at 853.3 eV is typical for NiS_x environments, but the Ni 2*p* core level cannot be used to distinguish between different NiS_x phases as their binding energies are almost identical [7]. In addition, a considerable contribution from oxidised nickel species is observed at 855.8 eV. This is expected as the samples were treated and handled in air. All three main $2p_{3/2}$ photoemission lines also result in satellite features, which give rise to a broad shoulder between 859 and 862 eV marked as “sat” in Supplementary Figure 4 (b), in agreement with values reported in the literature [8].

The S 2*p* core level in Supplementary Figure 4 (c) shows three different chemical states being present on the sample surface. Due to the asymmetry of the core levels giving rise to a considerable tailing towards the higher binding energy and the presence of several chemical species with varying line shapes a complete and robust peak fit analysis is not possible. However, to aid interpretation of the spectrum, symmetric Voigt peaks representing the main two NiS_x species are included in Supplementary Figure 4 (c). This analysis still delivers trustworthy binding energy positions of the main $2p_{3/2}$ features but does not fully describe their correct line shapes. The spectrum is dominated by a contribution with a $2p_{3/2}$ peak at 161.4 eV typical for NiS. An additional feature to higher binding energy, with a $2p_{3/2}$ position of 162.1 eV is representative of Ni_3S_2. The overall spectral shape as well as the reported binding energy positions of the NiS_x species agree well with previous reports in the literature [7,9]. The small feature above 168 eV is commensurate with the presence of sulfur-oxygen environments such as SO_3 and SO_4. From the sulfur spectrum it is clear that the NiS_x species observed in the Ni core level is predominantly NiS, with a small contribution from Ni_3S_2. Ni_3S_2 is easily oxidised to NiS in air as shown in Eq. S1:

$$\mathrm{Ni_3S_2} + \tfrac{1}{2}\mathrm{O_2} + \mathrm{H_2O} \rightarrow 2\mathrm{NiS} + \mathrm{NiO}\cdot\mathrm{H_2O} \qquad \text{[S1]}$$

This means that even if Ni_3S_2 is formed in the initial reaction, a subsequent conversion to NiS is expected.

The O 1*s* core level spectrum, as shown in Supplementary Figure 4 (d), confirms the observations of both Ni and S core levels. A considerable peak at 531.7 eV is typical of Ni hydroxide species. No lower binding energy features are observed, indicating the absence of extended nickel oxide species. The highest binding energy feature at around 533 eV is typical of water adsorbed on the sample surface, which is often observed on both Ni_xS_y as well as $Ni(OH)_2$ surfaces. The XPS data provides clear evidence that the surface of nickel is sulfidised after pre-treatment, and that the composition of the sulfidised nickel is a mixture of NiS or Ni_3S_2.

Supplementary Note 2 Osmotic effect and its influences

A potential problem associated with the future application of dual-membrane structures in PSA-RFBs is the osmotic effect, as identified in other RFB system[10]. When the battery is cycled with high concentrations of polysulfide anolyte, the large concentration difference between the anolyte and the intermediate solution would result in an osmotic water migration from the intermediate solution to the anolyte, diluting the anolyte and impeding reverse hydroxide transportation. One literature work in vanadium flow battery has proposed to balance the osmotic pressure in vanadium cells by adding a soluble draw solute such as 2-methylimidazole[11]. Similarly, we expect that the osmotic pressure in our dual membrane cell can be balanced by the addition of electrochemically-inert salt or molecules as draw solute into the middle buffer solution.

The electro-osmotic effect would also influence the water management of air-electrode [12,13], which consequently influences the hydroxide diffusion through the AEM.

During the charging process, water and oxygen is produced at the air electrode, and OH- moves from polysulfide side to air side simultaneously. Electro-osmotic drag causes the water to move from intermediate solution to the air electrode, causing the air electrode to be flooded. However, the influence of water flooding to the oxygen evolution reaction at air side during charging process is minimal as the main reactant is OH- from the aqueous phase, and the oxygen will spontaneously exit the cathode.

During the discharging process, the water is consumed at the air side and OH- moves from the air electrode to the polysulfide side. The electro-osmotic drag will further cause the water to move in the same direction as the OH-, i.e. from air side to polysulfide side. This potentially causes AEM drying out and lowering of its hydroxide conductivity. Therefore, during the operation we bubbled the oxygen through hydroxide solution so the oxygen flow could keep the cathode humidified. We didn't observe any reduction of performance caused by the AEM drying out.

Supplementary Note 3 Calculation of the power cost of storage

The power cost of storage (C_{power}, US\$/kW) of an RFB can be calculated as follows:

$$C_{power}\ (\frac{US\$}{kW}) = \frac{C_{stack}\ (\frac{US\$}{m^2})}{P_{peak}\ \left(\frac{mW}{cm^2} \times \frac{kW}{1000000\ mW} \times \frac{10000\ cm^2}{m^2}\right)}$$

Where, C_{stack} represents the cost of the stack (US\$/m^2), and P_{peak} represents the peak power density achieved during the polarisation measurements (mW/cm^2).

C_{power} of this alkaline PSA RFB and the previously reported state-of-the-art PSA RFB using acidic catholyte are calculated based on the peak power densities achieved during the polarisation measurements, and presented in Supplementary Table 4 and S5, respectively. Costs of the cell components are estimated by the bulk price of the corresponding material, which is obtained from the online supplier wherever possible [1]. The costs of the membrane (including both IEMs and SSE separator), the bipolar plate and the GDL are adopted from the projections by the US Department of Energy [2]. The cost of AEM is estimated as identical to the CEM, due to the lack of reporting for the AEM cost, which however should be cheaper than the CEM in the real case. The costs of other cell components, such

as the end plate, current collector plate and pipes, as well as the assembly costs are neglected in this calculation to simplify the discussion.

Supplementary Note 4 Calculation of the energy cost of storage

The energy (chemical) cost of storage (C_{energy}, US$/kWh) of a redox flow battery (RFB) can be calculated as follows:

$$C_{energy}\left(\frac{US\$}{kWh}\right)=\frac{C_{anolyte}\,(US\$)+C_{catholyte}\,(US\$)}{\bar{V}\,(V)\times I\left(\frac{mA}{cm^2}\times\frac{A}{1000\,mA}\right)\times A\,(cm^2)\times t\left(s\times\frac{h}{3600\,s}\right)\times\frac{kWh}{1000\,Wh}}$$

Where, $C_{anolyte}$ represents the cost of the anolyte (US$), $C_{catholyte}$ represents the cost of the catholyte (US$), $\bar{V}$ represents the average discharge voltage (V), I represents the discharge current density (mA/cm^2), A represents the active area of the cell (cm^2), and t represents the discharge duration (s).

C_{energy} of this alkaline PSA RFB and the previously reported state-of-the-art PSA RFB using acidic catholyte are calculated based on a total dissolved S content of 5 M, and presented in Tables S2 and S3, respectively. For both systems, it is assumed that the discharge voltage efficiency (i.e. average discharge voltage/OCV) is 70%[3]. The bulk prices of the chemicals used to prepare the electrolytes in both systems are obtained from the online supplier [1].

References

1 Fan, L., Wang, C. & Zhu, B. Low temperature ceramic fuel cells using all nano composite materials. *Nano Energy* **1**, 631-639, doi:https://doi.org/10.1016/j.nanoen.2012.04.004 (2012).

2 Fuentes, R. O. & Baker, R. T. Synthesis and properties of Gadolinium-doped ceria solid solutions for IT-SOFC electrolytes. *Int. J. Hydrogen Energy* **33**, 3480-3484, doi:https://doi.org/10.1016/j.ijhydene.2007.10.026 (2008).

3 Li, Z. *et al.* Air-Breathing Aqueous Sulfur Flow Battery for Ultralow-Cost Long-Duration Electrical Storage. *Joule* **1**, 306-327, doi:https://doi.org/10.1016/j.joule.2017.08.007 (2017).

4 Yu, M., Wang, C., Bai, Y., Wang, Y. & Xu, Y. Influence of Precursor Properties on the Thermal Stabilization of Polyacrylonitrile Fibers. *Polym. Bull. (Berlin)* **57**, 757-763, doi:10.1007/s00289-006-0629-9 (2006).

5 Adria Wilson, G. K., and Dimitrios Papageorgopoulos. Record 17007: Fuel Cell System Cost - 2017 (U.S. Department of Energy (Hydrogen and Fuel Cells Program)).

6 Gross, M. M. & Manthiram, A. Aqueous Polysulfide–Air Battery with a Mediator-Ion Solid Electrolyte and a Copper Sulfide Catalyst for Polysulfide Redox. *ACS Applied Energy Materials* **1**, 7230-7236, doi:10.1021/acsaem.8b01679 (2018).

7 Jiang, N. *et al.* Nickel sulfides for electrocatalytic hydrogen evolution under alkaline conditions: a case study of crystalline NiS, NiS2, and Ni3S2 nanoparticles. *Catalysis Science & Technology* **6**, 1077-1084, doi:10.1039/C5CY01111F (2016).

8 Legrand, D. L., Nesbitt, H. W. & Bancroft, G. M. X-ray photoelectron spectroscopic study of a pristine millerite (NiS) surface and the effect of air and water oxidation. *American Mineralogist* **83**, 1256-1265, doi:10.2138/am-1998-11-1214 (1998).

9 Buckley, A. N. & Woods, R. Electrochemical and XPS studies of the surface oxidation of synthetic heazlewoodite (Ni3S2). *Journal of Applied Electrochemistry* **21**, 575-582, doi:10.1007/bf01024844 (1991).

10 Hagemann, T. *et al.* An aqueous all-organic redox-flow battery employing a (2,2,6,6-tetramethylpiperidin-1-yl)oxyl-containing polymer as catholyte and dimethyl viologen dichloride as anolyte. *J. Power Sources* **378**, 546-554, doi:https://doi.org/10.1016/j.jpowsour.2017.09.007 (2018).

11 Yan, L. *et al.* Balancing Osmotic Pressure of Electrolytes for Nanoporous Membrane Vanadium Redox Flow Battery with a Draw Solute. *ACS Appl. Mater. Interfaces* **8**, 35289-35297, doi:10.1021/acsami.6b12068 (2016).
12 Eriksson, B. *et al.* Quantifying water transport in anion exchange membrane fuel cells. *Int. J. Hydrogen Energy* **44**, 4930-4939, doi:https://doi.org/10.1016/j.ijhydene.2018.12.185 (2019).
13 Moçotéguy, P., Ludwig, B., Beretta, D. & Pedersen, T. Study of the impact of water management on the performance of PEMFC commercial stacks by impedance spectroscopy. *Int. J. Hydrogen Energy* **45**, 16724-16737, doi:https://doi.org/10.1016/j.ijhydene.2020.04.139 (2020).
